# Development and Application of an iELISA for the Detection of Antibody against *Salmonella* Abortusequi

**DOI:** 10.1155/2023/1403180

**Published:** 2023-05-15

**Authors:** Kui Guo, Zenan Zhang, Yan Yang, Weiguo Zhang, Jinhui Wang, Shuaijie Li, Xiaoyu Chu, Wei Guo, Diqiu Liu, Yaoxin Wang, Zhe Hu, Xiaojun Wang

**Affiliations:** State Key Laboratory for Animal Disease Control and Prevention, Harbin Veterinary Research Institute, The Chinese Academy of Agricultural Sciences, Harbin, China

## Abstract

Equine abortus salmonellosis is a bacterial disease that causes high abortion rates in susceptible equids and therefore significant economic losses. Although the tube agglutination test (TAT) is a commonly used serological test for *S.* Abortusequi, it is not highly specific or sensitive, and the development of more sensitive, specific and rapid assays is therefore urgently required. In this study, an indirect enzyme-linked immunosorbent assay (iELISA) was developed for the specific detection of flagellum protein (FljB) antibodies against *S.* Abortusequi. Negative sera from horses in China (*n* = 1030) were used to establish the baseline for a negative population, and reference antisera positive against other viruses or bacteria were used to test the cross reactivity of the technique. The performance of the FljB iELISA was evaluated against that of the standard TAT, and was tested using field serum samples. The FljB iELISA assay was 8–16 times more sensitive than TAT. ROC analysis showed that the FljB iELISA was accurate, with an area under the curve (AUC) = 0.9943 (95% CI, 0.9815–1.000). The diagnostic sensitivity (DSe) of the FljB iELISA was 98.9% (95% Cl, 93.84%–100.00%), which was higher than that of TAT (DSe 38.6; 95% CI, 29.14%–49.08%). The diagnostic specificity (DSp) of the iELISA was 100.0% (95% CI, 95.82%–100.0%). When the 508 clinical samples were tested, the FljB iELISA had a positive detection rate of 51.38% (261/508, 95% CI, 51.24%–51.51%), which was higher than that of TAT (44/508). We also performed a serological survey for *S.* Abortusequi infection, using a series of samples collected from across eighteen provinces of China in 2021. The results showed that all provinces except Jiangsu had a certain number of cases, and the positive rates ranged from 0% to 96.9%, indicating the wide spread of *S.* Abortusequi in China. The abovementioned results suggest that the FljB iELISA developed in this study is rapid, sensitive, specific, and repeatable and is likely to be a suitable test for large-scale serological surveys for the detection and control of *S.* Abortusequi infection.

## 1. Introduction

Salmonellosis is one of the most important bacterial diseases in several animals and has caused huge economic losses in the pig [1], poultry [2], pigeon [3], and equine industries [4, 5]. *Salmonella enterica* subsp. *enterica* serovar Abortusequi (*S.* Abortusequi) is the most common causative agent of equine abortus salmonellosis and is well known as a host-adapted serovar that is associated with abortion in mares, neonatal septicemia, and multiple abscesses, orchitis and polyarthritis [4, 6–9]. Equine abortus salmonellosis was first described in 1893 in the United States. Although abortion caused by *S.* Abortusequi has been well controlled in the United States due to the strict breeding and health policies adopted in the 1950s, it has been frequently reported in Italy [10, 11], Croatia [8, 12], *Argentina* [13, 14], and Japan [6, 15, 16]. During the 1970s and 1980s, equine abortus salmonellosis was also commonly reported in China. Although the disease has rarely been reported since the 1980s, the high abortion rate associated with *S.* Abortusequi infection in equids has re-emerged over the last decade in China, the abortion rate in infected mares is between 30 and 100%. For example, during an abortion outbreak occurred in 2018 in a herd located in Inner Mongolia, China, 751 of 1051 pregnant mares aborted.

Currently, the gold standard for detecting carriers of *S.* Abortusequi is detection of the agent, but this is both time-consuming and laborious [17]. In addition, fecal detection can result in false negatives even in farms at which an outbreak of *S.* Abortusequi infection has occurred [18]. Serological analyses, such as the tube agglutination test (TAT) are a useful and important tool in diagnosis [8, 16]. However, the World Organisation for Animal Health (WOAH) reports that the TAT is not highly specific and may cross react with antibodies from other enterobacteria [19]. An iELISA based on lipopolysaccharide (LPS) was reported to be able to detect antibodies against *S.* Abortusequi with high sensitivity [20]. However, LPS-based iELISA may cross react with antibodies from other Gram-negative bacteria [21, 22], and no specific serological method capable of the specific identification of *S.* Abortusequi has been published to date. The development of a specific serological assay for the detection of *S.* Abortusequi is therefore of great importance.

The flagellin gene is commonly used as a serotype detection identifier for *Salmonella* [23–29]. In addition, flagellin protein was considered as a potential target candidate for the serological detection of *Salmonella*, since the protein is abundantly expressed, located on the surface of bacteria, and has good antigenicity [30–34]. *S.* Abortusequi lacks the phase (1) flagellar antigen encoded by FliC, and only expresses the phase (2) flagellar antigen encoded by FljB. Therefore, the aim of the present study was to develop a specific serological diagnostic method based on targeting antibodies against the FljB protein.

*S.* Abortusequi is a common serovar being able to cause equine abortus salmonellosis, however, the others three *Salmonella* (*S.* Typhimurium, *S.* Enteritidis and *S.* Dublin) may also cause this disease in equids in rare cases [35]. In order to develop a specific ELISA assay, we compared and analyzed the amino acid sequences of the FljB and/or the FliC flagellin proteins from different *Salmonella* serotypes and other members of the enterobacterales. A sequence fragment of *S.* Abortusequi FljB which can distinguish *S.* Abortusequi from *S.* Typhimurium, *S.* Enteritidis, *S.* Dublin, and other members of the enterobacterales, was tailored and then prepared as recombinant FljB (rFljB) protein in a prokaryotic expression system.

In this study, an iELISA assay based on the FljB protein was developed to specifically detect *S.* Abortusequi antibodies in horse serum. We then conducted a thorough analysis of the performance of the FljB iELISA and compared it to that of TAT by testing a large number of samples (Tables 1 and 2). Furthermore, we also performed a serological survey of *S.* Abortusequi in China using serum samples collected from the following eighteen Chinese provinces: Beijing, Chongqing, Guizhou, Gansu, Guangzhou, Hubei, Henan, Hebei, Jiangsu, Inner Mongolia, Jilin, Liaoning, Ningxia, Shandong, Sichuan, Shanxi, Tianjin, and Xinjiang. To our knowledge, this is the first time a large-scale serological survey of *S.* Abortusequi has been reported. Large-scale surveys such as this are necessary for the control and prevention of future equine abortus salmonellosis cases in China.

## 2. Materials and Methods

### 2.1. Serum Samples

A total of 3205 equine sera were used in this study (Table 1). One standard positive and one standard negative serum were collected from our laboratory for the development of iELISA. One thousand and thirty negative sera were collected from healthy horses in China and were used to determine the cut-off value. Three samples collected from our lab and verified by TAT as having High (High titer serum, 8×), Medium (Medium titer serum, 4×) or Low (Low titer serum, 1×) antibody concentrations, were used for testing the analytical sensitivity of iELISA. Fourteen reference antisera positive against other viruses or bacteria were selected for the analytical specificity test. Eighty eight clinical serum samples from horses, which were identified as infections with *S.* Abortusequi by bacteria isolation and 16S rRNA sequencing, and 88 samples from healthy horses, were used to perform the receiver operator characteristic (ROC) analysis. Comparison of the positive detection rates was performed by testing 508 clinical serum samples (335 samples from infected zones, 173 samples from healthy horses) using both TAT and iELISA. A further of 1472 serum samples were collected from 51 farms in eighteen provinces across the country in 2021 for the epidemiological investigation.

### 2.2. Isolation and Identification of *S*. Abortusequi

The methods for isolation and identification of *S*. Abortusequi were performed as our previously study described [5]. Briefly, vaginal swab liquid was plated on chromogenic *Salmonella* agar medium. After incubating at 37°C overnight, the obtained purple colonies were identified by the slide agglutination reaction of O- and H-antigens, and 16S rRNA sequencing. When the antigenic formula of the isolate serotyped was found to be 4, 12:-:*e*, *n*, *x*, and the result of 16S rRNA sequencing was determined to be *Salmonella*, the isolate was determined as *S*. Abortusequi, and the horse was thought to be infected with *S*. Abortusequi.

### 2.3. Preparation of Antigens

A total of 32 amino acid sequences of the FljB and/or FliC flagellin proteins from *S.* Abortusequi, other *Salmonella* serovars (Groups A–F) and other members of the enterobacterales were aligned using the MegAlign software (DNAStar, USA). The sequence of *S.* Abortusequi FljB which can distinguish *S.* Abortusequi from *S.* Typhimurium, *S.* Enteritidis and *S.* Dublin, was cloned into a bacterial expression plasmid pET28a vector and was expressed in *Escherichia coli* (*E. coli*) BL21 DE3 cells. Expression of the recombinant FljB (rFljB) protein in lysed *E. coli* DE3 cells was assessed by sodium dodecyl sulfatepolyacrylamide gel electrophoresis (SDS-PAGE), followed by Western blotting using anti-His specific antibody and *S.* Abortusequi-positive and -negative equine sera. The obtained hexahistidine fusion proteins were purified as previously described [36]. The purified protein was analyzed using SDS-PAGE on a 12% or 4–12% polyacrylamide gel. The concentration of protein was determined by using a Bicinchoninic acid (BCA) Protein Assay Kit (Novagen, Darmstadt, Germany). The purified protein with concentrations normalized to 1 mg/mL was used as the antigen in the FljB iELISA.

### 2.4. Establishment of FljB iELISA

The purified rFljB protein was used as the coating antigen to detect FljB antibodies in serum samples. For the iELISA, 96-well microtitre plates were coated with purified FljB antigen overnight at 4°C in 100 *μ*L PBS (pH 7.4). All washing steps were performed three times with washing buffer (PBS containing 0.1% Tween 20, PBST). The antigen-coated plates were blocked with PBS containing 5% skim milk for 1.5 h at 37°C. After washing, each serum was tested in duplicate, including standard positive and negative sera. Serum samples (diluted with PBS containing 5% skim milk) were incubated with coating antigen at 37°C for 1 h. The wells were washed three times with PBST, followed by incubation for 1 h at 37°C with HRP-labelled goat anti-horse IgG (Sigma-Aldrich). After incubation, the plate was washed again and incubated with freshly prepared TMB peroxidase substrate (GalaxyBio, Beijing, China) for 10 min at 37°C. The reaction was stopped by adding 2 M·H_2_SO_4_, and the optical density at the 450-nm wavelength (OD_450_) was measured using the VersaMax Microplate Reader (BioTek, Winooski, USA).

To develop the iELISA assay, several antigen coating concentrations, sera, and secondary antibody dilutions were applied using the checkerboard titration method. The 96-well microtitre plates were coated with purified FljB protein at 100 *μ*L per well, and diluted with coating buffer at 2, 1, or 0.5 *μ*g/mL. The positive and negative sera were diluted with PBS containing 5% skim milk to 1 : 200, 1 : 400 or 1 : 800. The secondary antibody was diluted by PBS containing 1% BSA to 1 : 5000, 1 : 10000 or 1 : 15000. Based on the ratio of positive to negative serum OD_450_ values (*P*/*N*), the antigen and serum concentrations at the maximum *P*/*N* ratio were used as the optimal reaction conditions for the subsequent ELISA assay. The means (*M*) and standard deviations (SDs) of the OD_450_ for 1030 negative controls were used to estimate the cut-off level. An OD_450_ value over *M *+* *3SD was considered to be positive.

### 2.5. Evaluation of FljB iELISA

To validate the specificity of the FljB iELISA, equine infectious anemia virus (EIAV), equine herpes virus (EHV-1, EHV-2, EHV-3, EHV-4, EHV-7), equine arteritis virus (EAV), and equine influenza viruses (EIV H7N7 and H3N8), *Streptococcus equi* (*S. equi*), *Escherichia coli* (*E. coli*), *S.* Typhimurium, *S.* Dublin, and *S.* Enteritidis antibodies were tested. *S.* Abortusequi positive serum was used as the positive control. The sensitivity of the iELISA was verified by testing serially two-fold-diluted serum, which was verified by TAT with the maximum detection dilutions of 8 (High titer serum, *H*), 4 (Medium titer serum, *M*), and 1 (Low titer serum, *L*). The detection limitations of the iELISA were determined based on the cut-off value. To evaluate the intra-assay reproducibility, positive serum samples at *H*, *M*, and *L* concentrations were tested in three replicates. To evaluate inter-assay repeatability, the same samples were assessed on three independent ELISA plates.

### 2.6. Western Blotting

Western blotting was performed as described previously [36]. Briefly, 0.25 *μ*g of purified rFljB protein or cell lysates expressed in *E. coli* BL21 DE3 was subjected to 12% or 4–12% SDS-PAGE. Following SDS-PAGE, the separated proteins were transferred onto nitrocellulose filter (NC) membranes. The membranes were blocked with PBS containing 5% skim milk (*w*/*v*) for 2 h and were incubated with then 25 ng/mL of anti-His antibody or a serum sample at a dilution of 1 : 50–1 : 1000 for 2 h at room temperature. The membranes were then incubated with anti-mouse or anti-horse IgG coupled to DyLight800 (KPL, Hemet, USA) at a dilution of 1 : 5000 for 1 h at room temperature. Protein was then detected using an Odyssey infrared imaging system (LI-COR, UK) at a wavelength of 800-nm.

### 2.7. Tube Agglutination Test (TAT)

To further evaluate the sensitivity of FljB iELISA and TAT, the same sera (Table 2) either infected with or negative for *S.* Abortusequi were tested using iELISA and TAT. TAT was performed as previously described [37].

### 2.8. Statistical Analysis

Statistical analysis was performed using receiver operator characteristic (ROC) curve analysis as a statistical tool for determining the diagnostic sensitivity and specificity in standard samples. The area under the ROC curve (AUC) was employed to assess the accuracy of FljB iELISA, TAT and Western blotting assays (an AUC of 1 indicates perfect discriminatory value; an AUC of 0.5 or less indicates no discriminatory value).

## 3. Results

### 3.1. Expression of Recombinant FljB Protein

**Figure 1:**
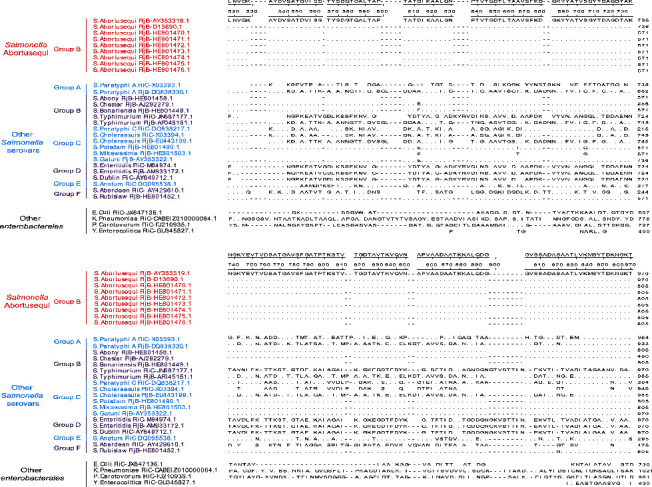
Amino acids sequence alignment of FljB and/or FliC from *S.* Abortusequi, other different *Salmonella* serotypes and other members of the enterobacterales.

**Figure 2:**
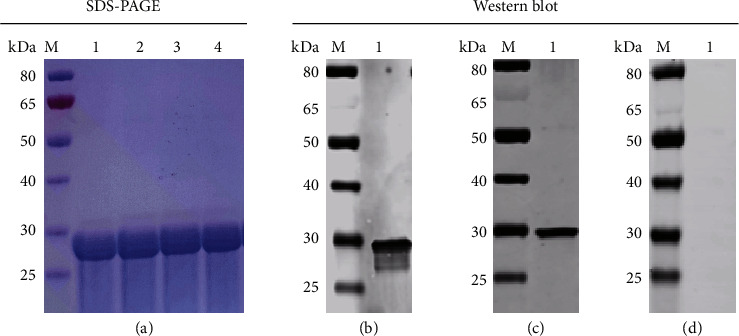
Purification and identification of recombinant FljB protein. (a) SDS–PAGE analysis of purified FljB protein. *M*, protein marker; lane 1–4, purified protein. (b) Western blotting detection of FljB using an anti-his tag antibody. (c) western blotting detection of FljB using *S.* Abortusequi positive serum. (d) Western blotting detection of FljB using *S.* Abortusequi negative serum.

The amino acids of the FljB proteins from the different *S.* Abortusequi strains were aligned with homologous sequences from different strains of FljB and/or FliC from different *Salmonella* serotypes or from other members of the enterobacterales. The results of the amino acid sequence analysis showed that the FljB (182–318aa) had a good conservatism with 100% identity between the 9 different strains of *S.* Abortusequi. The *S.* Abortusequi FljB (182–318aa) can distinguish *S.* Abortusequi from *S.* Typhimurium, *S.* Enteritidis, *S.* Dublin, and most *Salmonella* serotypes from Group A to Group F. Notably, the *S.* Abortusequi FljB also match FljB protein of seven strains of other *Salmonella* serovars. However, these *Salmonella* serovars have never been reported to cause abortion in horses (Figure 1). The sequence of the *S.* Abortusequi FljB gene (544–954 bp) was cloned into the pET28a vector, and expressed in *E. coli* BL21 DE3 cells. SDS-PAGE results revealed that the molecular weight of rFljB was approximately 29 kDa (Figure 2(a)). In Western blotting, anti-His tag antibody (Figure 2(b)) and *S.* Abortusequi-positive serum (Figure 2(c)) gave a positive reaction band at 29 kDa position. The negative control serum showed no reactivity with rFljB protein (Figure 2(d)). Subsequently, the purified rFljB protein was used to make coating antigen for the establishment of the ELISA.

### 3.2. The Establishment and Optimization of the FljB iELISA

The reaction efficiency of different amounts of FljB to serial dilutions of serum and secondary antibody was assessed using a checkerboard titration, and the highest *P*/*N* ratio value was obtained when 1 *μ*g/mL of FljB was used. Two dimensional titration matrix tests indicated that the optimal dilutions were 1 : 200 for serum and 1 : 10000 for secondary antibody. To establish the cut-off value, 1030 serum samples verified as negative using Western blotting were used as negative controls. Their resulting OD_450_ values showed an average absorbance of 0.077, with a standard deviation of 0.025. Therefore, the cut-off value of this iELISA was defined as 0.141 (*M* + 3SD). All the serum samples with OD_450_ values higher than 0.141 were determined as *S.* Abortusequi antibody positive, while if the OD_450_ values were less than or equal to 0.141, the sera were considered to be antibody negative.

### 3.3. Specificity, Sensitivity, and Reproducibility of Antibody Detection by the FljB iELISA

**Figure 3:**
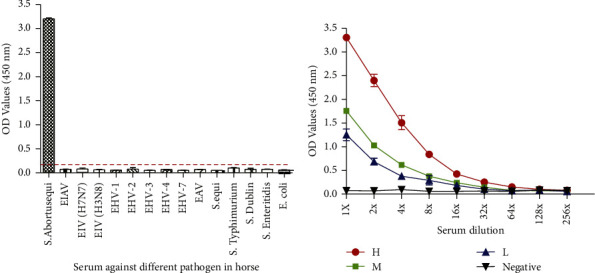
Analytical specificity and sensitivity of the FljB iELISA. (a) Test sera included a standard positive serum of *S.* Abortusequi and horse sera positive for various pathogens. (b) Two-fold serial dilutions of *S.* Abortusequi positive (*H*, *M*, and *L*) and negative sera were analyzed to determine the optimum dilution fold of serum samples tested using FljB iELISA. The error bars give the means of duplicate experiments.

The specificity of the detection by the FljB iELISA was investigated using a panel of antisera against EIAV, EHV-1, EHV-2, EHV-3, EHV-4, EHV-7, EAV, EIV (H3N8, H7N7), *S. equi*, *E. coli*, *S.* Dublin, *S.* Enteritidis, and *S.* Typhimurium. The OD_450_ values from all these serum samples were found to be below the cut-off value of 0.141 (Figure 3(a)). The above results showed that the FljB iELISA had a desirable specificity without serological cross-reactions with other reference antisera. The sensitivity of the detection by the FljB iELISA was investigated using sera at High (*H*, 8×), Medium (*M*, 4×), and Low (*L*, 1×) concentrations serum which had been verified by TAT. For *H*, *M*, and *L* concentrations of serum, positive values were still produced at dilutions of 64, 32, and 16, indicating the high sensitivity of the established FljB iELISA (Figure 3(b)). To test the reproducibility of iELISA, positive sera at *H*, *M*, and *L* concentrations and negative serum samples were tested with three replicates in three independent ELISA assays. The mean OD_450_ values and standard deviations of the tests were 0.071 ± 0.007 for negative samples and 3.323 ± 0.02, 1.729 ± 0.038 and 1.211 ± 0.08 for positive sera at *H*, *M*, and *L* concentrations, respectively, demonstrating the reliability of the FljB iELISA.

### 3.4. Comparison of Diagnostic Sensitivity (DSe) and Diagnostic Specificity (DSp) of ELISA, TAT and Western Blotting

**Figure 4:**
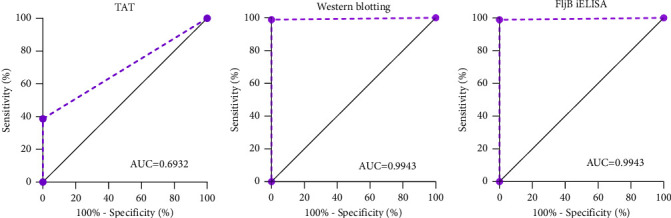
ROC analysis for FljB iELISA, TAT and western blotting. Overlay of all results of the ROC (receiver operating characteristics) analysis for all samples (overall) and for TAT (a), western blotting (b) and FljB iELISA (c), separately, with the corresponding area under the ROC curve (AUC). The true infection positives (*n* = 88) and true negatives (*n* = 88) were used as reference populations. The ROC plots the true positive rate (sensitivity) against the false positives (100-specificity).

The DSe was calculated for each test based on the results of 88 horse serum samples infected with *S.* Abortusequi infection, which had been taken from aborted horses confirmed as positive through isolation of *S.* Abortusequi (Table 2, Farms 1–3). For the evaluation of DSp, serum samples from healthy horses were used (Table 2, Farm 4). The performances of the three methods were then compared. The AUCs of TAT, Western blotting and FljB iELISA were 0.6932 (95% CI, 0.6143–0.7721), 0.9943 (95% CI, 0.9815–1.000) and 0.9943 (95% CI, 0.9815–1.000), respectively (Figures 4(a)–4(c)). TAT detected antibodies to *S.* Abortusequi in 34 (38.6%) of 88 serum samples from *S.* Abortusequi-infected horses, with low sensitivity but high specificity (100%). According to the AUC index, 69.32% of the samples were correctly classified (Figure 4(a)). Western blotting was able to detect *S.* Abortusequi antibodies with 98.9% (87/88) DSe and 100% (88/88) DSp (Figure 4(b)). The FljB iELISA DSe and DSp were consistent with those from Western blotting (Figure 4(c)).

### 3.5. Evaluation of FljB iELISA and TAT for *S.* Abortusequi Antibody Detection

**Figure 5:**
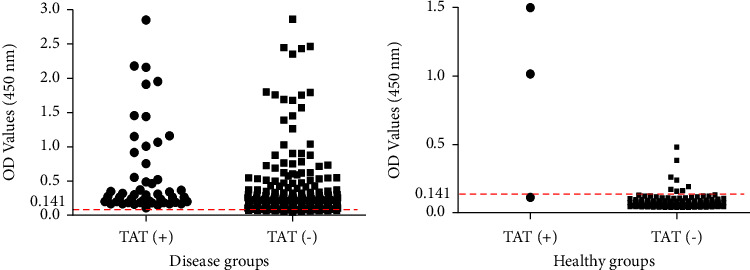
Statistical FljB iELISA OD value distribution based on the TAT results of serum samples from infected horses (a) and healthy horses (b). Horizontal line represents the cut-off value (0.141). Antisera were obtained from 9 different farms or regions in China.

The iELISA was applied in order to detect antibodies against *S.* Abortusequi from 508 field serum samples. Both subgroups of 335 clinical samples from infected zones (Table 2, Farms 5–8) and 173 samples from healthy horses (Table 2, Farms 9–13) were assessed. The total positive detection rates of the iELISA and TAT in serum samples from infected zones were 74.9% (251/335) and 12.2% (41/335), respectively. The positive detection rates of the FljB iELISA and TAT in healthy horse samples were 5.8% (10/173) and 1.7% (3/173), respectively. The forty-one samples from infected horses that tested positive in the TAT all also tested positive in the FljB iELISA (Figure 5(a)). One of the three samples from healthy horses that resulted in positive in the TAT but tested negative in the FljB iELISA (Figure 5(b)), was verified using Western blotting not to react with FljB protein (data not shown). Two hundred and eighteen TAT-negative but FljB iELISA-positive samples were verified as being able to react with rFljB protein using Western blotting (data not shown).

### 3.6. Serological Survey of *S.* Abortusequi in China

During 2021, a total of 1472 horse serum samples from 51 farms in eighteen Chinese provinces were tested, with most of the samples were originating from Inner Mongolia. Samples from seventeen provinces, with the exception of Jiangsu, were found to be positive (Table 3). A total of 480 *S.* Abortusequi-positive sera were detected in 2021. The majority of positive samples were from Shandong, Hebei, Inner Mongolia and Xinjiang.

## 4. Discussion

Equine abortus salmonellosis has occurred frequently in recent years and has caused serious economic losses to the equine industry in China [4, 5]. Therefore, the development of reliable serum diagnostic methods is essential to achieve the most efficient *S.* Abortusequi antibody detection. In earlier studies, LPS was used in an ELISA for the detection of *S.* Abortusequi, but the specificity of this technique needs to be further evaluated [20], and some studies showed that LPS can have a cross-reactivity problem with other Gram-negative bacteria [21, 22]. The previous researchers developed an ELISA using recombinant FliC flagellin for the detection of *S.* Enteritidis, and demonstrated that it had a good specificity and did not cross react with other bacteria[32]. In this study, we found that the *S.* Abortusequi FljB flagellin protein (182–318aa) is suitable as an iELISA candidate antigen to distinguish *S.* Abortusequi from *S.* Typhimurium, *S.* Dublin, *S.* Enteritidis, and most *Salmonella* serotypes from Groups A to F (Figure 1). Although the *S.* Abortusequi FljB (182–318aa) shares 99.6–100% homology with other seven *Salmonella* serovars with both the formula *H*: *e*, *n*, *x* (*S.* Abony, *S.* Chester and *S.* Bonariensis in Group B, *S.* Gatuni in Group C, *S.* Rubislaw in Group F) and *H*: *e*, *n*, *z* (*S.* Potsdam and *S.* Mikawasima in Group C), there have been no reports that these seven *Salmonella* serovars are able to cause infection in equines. Therefore, the FljB iELISA would be very helpful for clinical disease diagnosis.

In this study, we evaluated the performance characteristics of the newly developed FljB iELISA, including the diagnostic sensitivity and specificity. The technique was tested using positive and negative sample sera collected from animals with known health status, and showed a DSe and DSp of 98.9% (87/88) and 100% (88/88), respectively. The DSp of TAT was consistent with that of the FljB iELISA (100%), but the DSe of TAT was only 38.6%, lower than that of the FljB iELISA. The serum samples which tested negative in the TAT but positive in the iELISA were further verified using Western blotting, and all serum samples showed a positive reaction with rFljB protein. The only horse serum sample infected with *S.* Abortusequi but giving a negative result by iELISA (the TAT result was also negative), was further tested using Western blotting, and bacterial isolation analysis was also conducted on simultaneously collected plasma samples from this horse. Interestingly, the Western blotting result also showed that the serum sample was not able to react with rFljB protein, which was consistent with the FljB iELISA result. More surprisingly, a large number of *S.* Abortusequi bacteria were isolated from the plasma. Therefore, although the horse was sick, the serum samples were not tested positive by the Western blotting and FljB iELISA analyses. We therefore presume that this horse had not yet produced any antibodies against *S.* Abortusequi, or had produced low level antibodies that could not be detected.

We further conducted a thorough analysis of the performance of the FljB iELISA compared to that of TAT using clinical samples. A total of 335 clinical samples from infected zones (Table 2, Farms 5–8) and 173 samples from healthy horses (Table 2, Farms 9–13) were tested. We found that the positive detection rates of FljB iELISA were 74.9% (251/335) and 5.8% (10/173) in samples from infected and healthy serum samples, respectively, which were much higher than those from TAT. In order to determine whether the equivocal serum samples were positive or negative, all samples were further verified by Western blotting. Western blotting results showed that the only sample testing positive in the TAT but negative in the iELISA was not able to react with *S.* Abortusequi rFljB protein, *S.* Typhimurium, *S.* Enteritidis and *S.* Dublin FljB and/or FliC flagellin proteins (data not shown). The equivocal serum samples from infected or healthy horses (TAT negative but iELISA positive) were also further verified by Western blotting. A specific band confirming the presence of the *S.* Abortusequi rFljB protein was obtained from each serum (data not shown), demonstrating that the FljB iELISA results were reliable.

In order to investigate the prevalence of equine abortus salmonellosis in China, we also performed a serological survey using FljB ELISA to test for the presence of *S.* Abortusequi antibodies in 1472 serum samples collected from 51 farms in eighteen Chinese provinces in 2021. The results showed that the seroprevalence rate ranged from 0% to 96.9% (95% CI, 96.81%–96.99%). Seroprevalence rates of 96.9% (95% CI, 96.81%–96.99%), 67.0% (95% CI, 66.47%–67.53%), 49.6% (95% CI, 49.49%–49.71%) and 39.6% (95% CI, 36.50%–39.50%) were observed in Shandong, Hebei, Inner Mongolia, and Xinjiang, respectively, which is consistent with the number of local outbreaks of equine abortus salmonellosis reported from these provinces. The results of the serological survey also revealed that *S.* Abortusequi is widely spread in China (Table 3). This study represents the first large-scale serological survey of *S.* Abortusequi and is crucial for understanding the prevalence of equine abortus salmonellosis in China.

## Figures and Tables

**Table 1:** Serum samples employed in this study.

Purpose	Amounts	Name/description	Sample source
Development of iELISA	2	Standard positive and negative serum	Stored in our lab

Cut-off value determination	1030	Clinical healthy equine sera samples verified as negative by TAT and western blotting	Farms in China

Analytical specificity	6	Reference sera against equine herpesvirus 1 (EHV-1), 2 (EHV-2), 3 (EHV-3), equine influenza virus (EIV-prague and EIV-kentucky), and equine arteritis virus (EAV)	NVSL, USA
	8	Sera against equine infectious anemia virus (EIAV), equine herpesvirus (EHV-4 and EHV-7), *Streptococcus equi* (*S. equi*), *S.* Dublin, *S.* Typhimurium, *S.* Enteritidis, and *Escherichia coli*	Stored in our lab

Analytical sensitivity	3	Serums samples verified by TAT as having high (8×), medium (4×) and low (1×) antibody concentrations	Stored in our lab

Diagnostic sensitivity and specificity analysis	176	88 collected from the horses infected with *S.* Abortusequi in farms 1–3	Farms in China
		88 collected from the healthy horses in farm 4	

Comparison of positive detection rates between TAT and iELISA	508	335 from infected horses in farms 5–8	Farms in China
		173 samples from healthy horses in farms 9–13	

Serological surveillance	1472	1472 from 51 farms in 18 provinces	Farms in China

NVSL is an abbreviation for “National Veterinary Services Laboratories, Inc.” in USA.

**Table 2:** Comparison of the performance of FljB iELISA and TAT assays.

Location	Source of serum	Clinical symptoms	Vaginal swab	Pathogen identification	Serum samples	*TAT/iELISA*				*Positive detection rate (%) (95% CI)*	
						(+/+)	(+/−)	(−/+)	(−/−)	TAT	FljB iELISA
Farm 1	Mares	Abortion	43	*S.* Abortusequi	43	14	0	28	1	29.3 (27.56–31.04)	97.6 (97.49–97.41)

Farm 2	Mares	Abortion	20	*S.* Abortusequi	20	5	0	15	0	25.0 (21.33–28.68)	100.0 (100)

Farm 3	Mares	Abortion	25	*S.* Abortusequi	25	15	0	10	0	60.0 (57.57–62.43)	100.0 (100)

Farm 4	Mares	Normal	88	—	88	0	0	0	88	0	0

Farm 5	Mares	Abortion	5	*S.* Abortusequi	146	10	0	102	34	6.8 (6.47–7.13)	76.7 (76.43–76.97)

Farm 6	Mares	Abortion	12	*S.* Abortusequi	65	20	0	26	19	30.8 (29.64–31.96)	70.8 (70.06–71.54)

Farm 7	Mares	Abortion	6	*S.* Abortusequi	57	5	0	46	6	8.8 (7.87–9.13)	89.5 (89.16–89.84)
	Foals	Arthroncus	7	*S.* Abortusequi	43	3	0	21	19	7.0 (5.88–8.12)	55.8 (54.30–57.30)

Farm 8	Mares	Abortion	4	*S.* Abortusequi	24	3	0	15	6	12.5 (9.97–15.03)	75.0 (73.23–76.77)

Farm 9	Mares	Normal	—	—	50	0	1	0	49	2 (1.46–2.54)	0

Farm 10	Stallion	Normal	—	—	16	0	0	0	16	0	0

Farm 11	Mares	Normal	—	—	26	0	0	1	25	0	3.8 (2.39–5.21)

Farm 12	Mares	Normal	—	—	57	2	0	5	50	3.5 (2.88–4.12)	12.3 (11.24–13.36)

Farm 13	Stallion	Normal	—	—	24	0	0	2	22	0	8.3 (6.14–10.46)

**Table 3:** Serological surveillance of equine abortus salmonellosis in China in 2021.

Sample source (province)	No. of farms	No. of samples	No. of positive	Prevalence (%) (95% CI)
Shandong	2	65	63	96.9 (96.81–96.99)
Hebei	2	100	67	67.0 (66.47–67.53)
Inner Mongolia	17	607	301	49.6 (49.49–49.71)
Xinjiang	3	50	19	38.0 (36.50–39.50)
Chongqing	1	20	3	15.0 (11.77–18.33)
Gansu	2	20	2	10.0 (7.21–12.79)
Shanxi	2	50	4	8.0 (6.98–9.02)
Henan	3	50	4	8.0 (6.98–9.02)
Guangzhou	1	50	3	6.0 (5.10–6.90)
Liaoning	4	50	3	6.0 (5.10–6.90)
Ningxia	1	50	3	6.0 (5.10–6.90)
Hubei	1	50	2	4.0 (3.25–4.75)
Sichuan	6	50	2	4.0 (3.25–4.75)
Guizhou	2	50	1	2.0 (1.46–2.54)
Beijing	1	50	1	2.0 (1.46–2.54)
Jilin	1	50	1	2.0 (1.46–2.54)
Tianjin	1	60	1	1.7 (1.28–2.12)
Jiangsu	1	50	0	0 (0)
Total	51	1472	480	32.6 (32.55–32.65)

## Data Availability

The data used to support the findings of this study are included within the article.
